# Risk Factors and Diagnosis of Advanced Cutaneous Squamous Cell Carcinoma

**DOI:** 10.5826/dpc.11S2a166S

**Published:** 2021-09-01

**Authors:** Gabriella Brancaccio, Giulia Briatico, Cristina Pellegrini, Tea Rocco, Elvira Moscarella, Maria Concetta Fargnoli

**Affiliations:** 1 Dermatology Unit, University of Campania “Luigi Vanvitelli”, Naples, Italy; 2 Dermatology, Department of Biotechnological and Clinical Sciences, University of L’Aquila, L’Aquila, Italy

**Keywords:** advanced cutaneous squamous cell carcinoma, risk factors, prognostic factors, recurrence, metastasis

## Abstract

Cutaneous squamous cell carcinoma (cSCC) is the second most common cancer affecting humans. The combination of the increasing incidence and high mortality in advanced stages of the disease, defines cSCC as an emerging public health problem. Advanced disease includes metastatic and locally advanced cSCC. Metastatic disease refers to the presence of locoregional metastasis (in transit or to regional lymph nodes) or distant metastasis. Locally advanced disease has been defined as non-metastatic cSCC that is unlikely to be cured with surgery, radiotherapy, or combination treatment. While metastatic cSCC is easily diagnosed, locally advanced disease lacks consensus definition and diagnosis is made after multidisciplinary board consultation. Identifying patients with aggressive cSCC at highest risk for relapse may prevent the occurrence of advanced disease. Prognostic factors suggested by most guidelines include tumor diameter (>2 cm), localization on temple/ear/lip/area, thickness (>6 mm), or invasion beyond subcutaneous fat, poor grade of differentiation, desmoplasia, perineural invasion, bone erosion, immunosuppression, undefined borders, recurrence, growth rate, site of prior radiotherapy, and lymphatic or vascular involvement. Although risk factors associated with worse outcomes are well known, there is still a gap of knowledge on the precise risk of each factor taken individually. The aim of this review is to summarize cSCC prognostic factors and encompass the various staging systems to guide management and follow-up in cSCC patients at higher risk for local recurrence and metastasis. Finally, we describe the hallmarks of the advanced disease. Advanced cSCC diagnosis should be made by a multidisciplinary board considering patients’ performance status and disease characteristics.

## Introduction

Cutaneous squamous cell carcinoma (cSCC) is considered the second most common non melanoma skin cancer after basal cell carcinoma (BCC). Evidence indicates however that the incidence is still underestimated. Contrary to the reported BCC:SCC ratio of 4:1, a ratio of 1:1 was observed in the US Medicare fee-for-service Population from 2006 to 2012 [1], and an overall 263% increase in the incidence rate of SCC was observed from 1976 to 2010 in a population cohort from Minnesota [2].

cSCC is also regarded as the second most frequent cause of death due to skin cancer after melanoma, although, on a population-based scale, the absolute number of deaths from cSCC equals that of melanoma [3]. Despite the overall favorable clinical outcome of low risk cSCC, there is a subset of cSCCs that tends to recur and metastasize exhibiting a more aggressive course. The rate of recurrence varies from 2.7% [4] to 4.6% [5], as reported in 2 large studies including 653 and 985 patients with cSCC followed for approximately 10 years, respectively. The rate of metastases ranges from 1.2% to 4%, with 2.1% disease-specific death [5]. Because of the increasing incidence related to the aging population and the high mortality in advanced disease, cSCC is increasingly emerging as a public health problem.

The term “advanced cutaneous squamous cell carcinoma” defines cSCCs that are no longer amenable to surgery and/or radiotherapy (RT) and are eligible to anti PD-1 treatment [6]. The introduction of this class of drugs has raised awareness on advanced cSCC that was previously under-recognized and under-treated because of its poor prognosis. This draw attention towards the need for a clear and shared definition of advanced cSCC. However, the difficult management of advanced cSCCs requires that clinical and scientific efforts should be directed to prevent the occurrence of advanced disease. Risk assessment is therefore particularly important to identify the few cSCCs with a high risk of local recurrence or metastasis among all other low-risk tumors. High-risk cSCC should not turn into the advanced or metastatic form if properly managed with adequate surgery, follow-up, and adjuvant therapy. A thorough clarification of cSCC characteristics associated with poor prognosis is urgently needed, as it is crucial factor in guiding multidisciplinary discussions on an adequate management strategy.

### Classification and Staging of cSCC

The WHO classification of skin tumors identifies several histologic variants of cSCC which have important implication for management and prognosis [7]. Among invasive cSCC, keratoacanthoma and verrucous SCC are considered low-grade variants as they have little, if any, metastatic potential, while acantholytic, spindle cell, adenosquamous, and clear cell SCC are characterized by a more aggressive behavior and worse prognosis [7].

The 8^th^ edition of the TNM classification of malignant tumor (TNM8) was published in 2017, with a version from both the American Joint Committee on Cancer (AJCC) [8] and the Union for International Cancer Control (UICC) [9]. UICC and AJCC work closely together and, in most instances, the TNM version of each organization is the same or very similar. Unexpectedly, AJCC limited its TNM8 edition to the staging system for cSCC of the head and neck and did not provide a staging system for cSCC of the trunk and limbs. In comparison, UICC TNM8 provides 2 chapters for skin carcinoma: one covering the primary sites on the head and neck and one covering the trunk and limbs. Overall, the 2 chapters in UICC 8^th^ and AJCC 8^th^ ed. for cSCC TNM of head and neck are essentially identical except for the definition of perineural involvement (Tables 1–4) [9,10].

The T subcategory is defined by the clinical diameter and deep invasion of the primary tumor (with thresholds of 2 and 4 cm for clinical diameter and 6 mm as limit for deep invasion) and by perineural invasion or bone erosion as parameters of upgrade to T3 or T4a/b. However, the T2 subcategory comprises a wide range of tumors, some of them associated with poor prognosis [6]. The Brigham and Women’s Hospital (BWH) classification system for the T stage was developed to better correlate higher tumor stages with poor outcomes, as an effort to more accurately separate high-risk from low-risk tumors [11]. In detail, this classification system identifies poor differentiation, perineural invasion, invasion beyond subcutaneous tissue and diameter ≥2 cm as risk factors associated with worse prognosis and provides a quantifiable risk value according to the number of identified risk factors. Thus, T2 tumors are stratified into a low-risk T2a stage (with one of the above risk factors) with 16% of these patients accounting for all SCC-related events (recurrence, nodal metastasis and/or death) and a high-risk T2b with tumors combining 2–3 risk factors and accounting for 64% of all SCC-related events. T3 stage includes tumors combining all 4 risk factors, as well as those with bone invasion.

The N subcategory is differently addressed in the 2 chapters of the UICC classification system (skin carcinoma of the head/neck and carcinoma of trunk/limbs) [9]. Unlike cSCC of trunk and limbs, cSCC of the head and neck incorporates extranodal extension and laterality into its staging criteria.

The combination of T, N, and M categories defines the stage of the tumor, with UICC and AJCC as the most widespread and used staging systems (Table 5). Noteworthy, stage III in both UICC and AJCC staging systems includes cSCC, with or without nodal involvement, and stage IV includes cases with or without distant metastasis. These classifications seem to equate patients with advanced disease but with different characteristics. Likewise, both UICC and AJCC staging systems do not encompass all the potential risk factors for a worse prognosis of cSCC (eg location and differentiation) whereas a combination of prognostic factors should better guide management of cSCC in the multidisciplinary board.

### Prognostic Factors in cSCC

**Figure 1:**
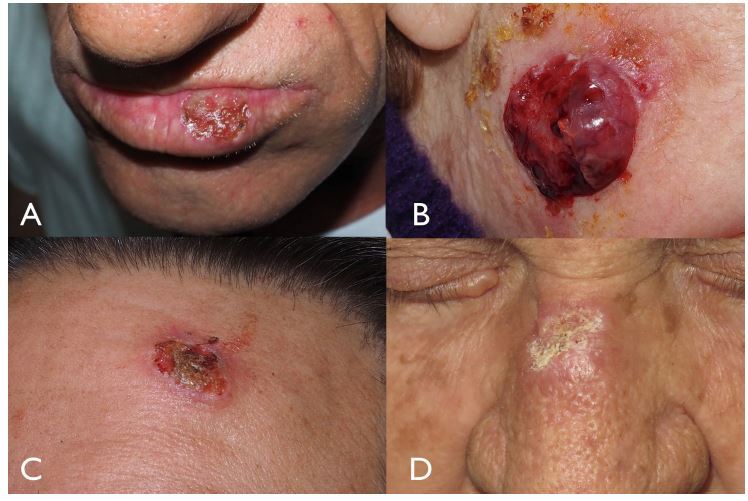
High-risk cutaneous squamous cell carcinoma (cSCC). (A) cSCC on the lower lip of a male aged 49, with high-risk features as location, thickness (4 mm), poor differentiation, and perineural invasion. Despite the small size, the patient developed nodal metastasis after 6 months from primary excision and adjuvant radiotherapy. (B) cSCC on the cheek of a female aged 77, defined as very high-risk according to NCCN guidelines: thickness 11.0 mm, > 4 cm in diameter, poor differentiation. (C) cSCC on the forehead of a patient aged 58, at high-risk because of poor differentiation, presence of perineural invasion, diameter > 2 cm. (D) cSCC on the nose of a patient aged 83 characterized by poor differentiation and perineural invasion.

Among the most authoritative guidelines for diagnosis and management of cSCC, EADO [12] and NCCN [13] guidelines, address the differentiation of high-risk from low-risk tumors (Table 6) (Figure 1). Prognostic high-risk factors proposed by EADO include tumor diameter (>2 cm), location on temple/ear/lip/area, thickness (>6 mm), or invasion beyond subcutaneous fat, poor grade of differentiation, desmoplasia, microscopic, symptomatic, or radiological perineural invasion, bone erosion, and immunosuppression. NCCN guidelines add as prognostic factors positive borders, primary vs recurrent, growth rate, site of prior radiotherapy, lymphatic, or vascular involvement and, very recently, subclassified cSCC into high-risk and very high-risk. Any high-risk factor places the patient in the high-risk category.

Risk factors associated with poor prognosis in cSCC can be classified as intrinsic (tumor-related) and extrinsic (patient- and physician-related) [6].

#### Tumor-Related Prognostic Factors

##### Size

Tumor diameter > 2 cm is the risk factor most associated with disease-specific death, with a 3-fold greater risk of recurrence and a 6-fold greater risk of metastasis [8]. A meta-analysis that included 36 studies and 17,248 patients set at 4 cm the most specific cutoff for determining the risk associated with tumor size [14]. Other studies have confirmed worse outcomes in T2 than in T1 tumors, but revealed that beyond the 2 cm cut-off, the effect on disease free survival (DFS) becomes smaller [15][16].

##### Location

cSCC of the ear, temples and lips are associated with higher risk of recurrence and metastasis compared to other body regions. The risk of nodal metastasis is 5-fold greater for cSCCs on the vermilion lip compared with those on the cutaneous lip. The absence of subcutaneous fat in the vermilion lip might allow quicker tumor access to the rich lymphovascular space of muscle leading to a greater metastatic potential [17]. Also, hands, feet, pretibial and anogenital area are considered location at risk independent of the size.

##### Depth of invasion

Concerning thickness, the cut-off to differentiate low-risk versus high-risk cSCC has been set at 6 mm, and it is unanimously reported by all the available staging systems, guidelines and meta-analyses [13,14,18] When the depth of cSCC is reported as tumor invasion level, referring to the deepest tissue plan involved, the considered cut-off is the invasion beyond subcutaneous fat. Bone invasion is mentioned in many guidelines as an independent prognostic factor, as its presence upgrades to T3 (minor erosion) or T4 (gross invasion) according to the AJCC 8^th^ ed. staging system. However, it is comprised, by definition, in the tumor invasion level.

##### Perineural invasion

The impact of perineural invasion as risk factor for negative outcomes is the most well characterized. Many single institution reviews report a strong association with disease recurrence [15,17,19,20] and the meta-analysis by Thompson et al [14] confirmed a statistically significant association with disease-specific death and disease recurrence, with a risk ratio of 4.3 for disease recurrence. Thus, the AJCC 8^th^ ed. upstages thin cSCCs to T3 if a nerve greater than 0.1 mm in caliber is involved [18].

##### Poor differentiation

The histologic grading system proposed by Broder in 1921 identifies 4 grades of differentiation according to the percentage of well-differentiated cells in the tumor tissue (Grade 1: 75% of well-differentiated cells; Grade 2: 50% of well-differentiated cells; Grade 3: 25% to 50% of well-differentiated cells; Grade 4: <25% of well-differentiated cells) [21]. Tumors are also classified into well-differentiated, moderately-differentiated, and poorly-differentiated according to the presence of clear keratinization, horn pearls, and other classic histologic features of cSCC, or the difficulty to determine a keratinocyte lineage [22]. The grade of histologic differentiation is not taken into account by the 8^th^ edition of UICC and AJCC staging systems but remains a high-risk factor in the BWT system, NCCN, and EADO guidelines [6,11,13].

##### Desmoplasia

Desmoplastic cSCC is an aggressive histologic variant of cSCC characterized by narrow cords of cells and large amounts of extracellular stroma and often by perineural and perivascular invasion. Recurrence rate and metastatic potential are 10-fold and 6-fold higher than other cSCC variants, respectively [23].

##### Growth rate

It has been observed that a growth rate > 4 mm/month in the long axis of the tumor is associated with poor prognosis and a greater risk of lymph node metastasis [24]. However, only NCCN guidelines identify rapidly growing tumor among risk groups for local recurrence, metastases, or death from disease [13].

#### Extrinsic Prognostic Factors (Patient- and Physician-Related)

The role of extrinsic risk factors is more difficult to analyze, as features such as patient’s request to limit the extent of surgery or physician’s expertise in the treatment are impossible to standardize and systematically compare. However, in clinical practice, extrinsic factors have the greatest impact on the natural history of the tumor, as they may turn an early tumor with clinical and histological low-risk features into a cSCC with a high-risk of recurrence and worse outcome, namely an advanced cSCC.

##### Positive margins and recurrent disease

EADO guidelines do not include recurrence in the list of high-risk cSCC prognostic factors as it can be considered as the result of positive margins. Positive margins correspond to a residual tumor, which has potential for recurrence *a priori*. When initial removal is incomplete, cSCC is more likely to recur, mostly locally and less frequently in regional lymph nodes [6]. In order to obtain optimal tumor clearance in cSCC < 2 cm in size, EADO suggests 5 mm clinically tumor-free margins, while in cSCC >2 cm in size suggested margins are 6–10 mm [6]. However, involved borders after the initial excision often derive from subclinical infiltration in sun-damaged skin [25]. Recurrent cSCC are twice as likely to recur after excisional surgery when compared with primary tumors [25].

##### Site of prior radiotherapy or chronic inflammatory process

cSCCs arising from a leg ulcer, burn scar, radiation dermatitis, discoid lupus, and other chronic wounds have a reported metastatic risk of 26% [26]. This risk factor is only listed by the NCCN guidelines.

##### Immunosuppression

The incidence of cSCC in immunosuppressed individuals has been estimated to be 64 to 250 times higher than in the general population. The cumulative incidence of cSCC increases progressively with duration of immunosuppression and tumors developed in this setting show a more aggressive behavior [27,28].

##### Comorbidities and patient’ preferences

Comorbidities represent one of the major obstacles to surgery, which is the first-line therapy of cSCC. Furthermore, in the case of high-risk tumors located on the head and neck area, primary excision can be often destructive, leading the patient to refuse the treatment. Patient’s request to limit extent of surgery is indeed an additional, substantial risk factor.

### Managing Prognostic Factors - Practical Implications

Although numerous risk factors associated with worse outcomes have been identified, there is still a gap of knowledge on the precise risk of each factor individually. Combination of 2 or more factors is considered to significantly increase the risk of poor outcome. EADO guidelines recommend considering the variations of patient- and tumor-related characteristics when assessing the level of overall prognostic risk [6]. However, the decision still relies on the physician’ expertise and opinion, as neither a nomogram nor a scoring system are available yet to define which cSCC would deserve adjuvant RT or a closer follow-up program.

Adjuvant RT is offered as part of clinical practice in many medical centers for patients with high-risk cSCC, particularly for tumors with perineural invasion. However, there is still a lack of significant evidence, including randomized controlled trial data, showing a clear benefit of adjuvant RT in this setting [12].

There is no standardized follow-up schedule for patients with cSCC due to the lack of randomized controlled trials. Patients with high-risk cSCC should be followed up every 3–6 months for the first 2 years, and every 6–12 months for years 3–5, and annually thereafter [13]. Lymph node ultrasound should be performed every 3–6 months in the first 2 years depending on risk stratification. Again, as the independent prognostic effect of high-risk factors has not been consistently defined, EADO guidelines advice individual risk assessment to guide follow-up decisions [12].

### Diagnosis of Advanced Cutaneous Squamous Cell Carcinoma

Advanced cSCC is classified as metastatic cSCC or locally advanced cSCC.

Metastatic disease includes locoregional metastasis (in transit or to regional lymph nodes) or distant metastasis which are easily diagnosed by imaging (stage III and IV according to AJCC/UICC 8^th^ ed.]. Noteworthy, the 8^th^ ed. of AJCC/UICC staging system does not include the presence of in-transit metastases. The prognostic role of satellite and in transit metastasis in cSCC has been recently investigated by Xu et al [29] who found a significant association with worse overall survival.

**Figure 2:**
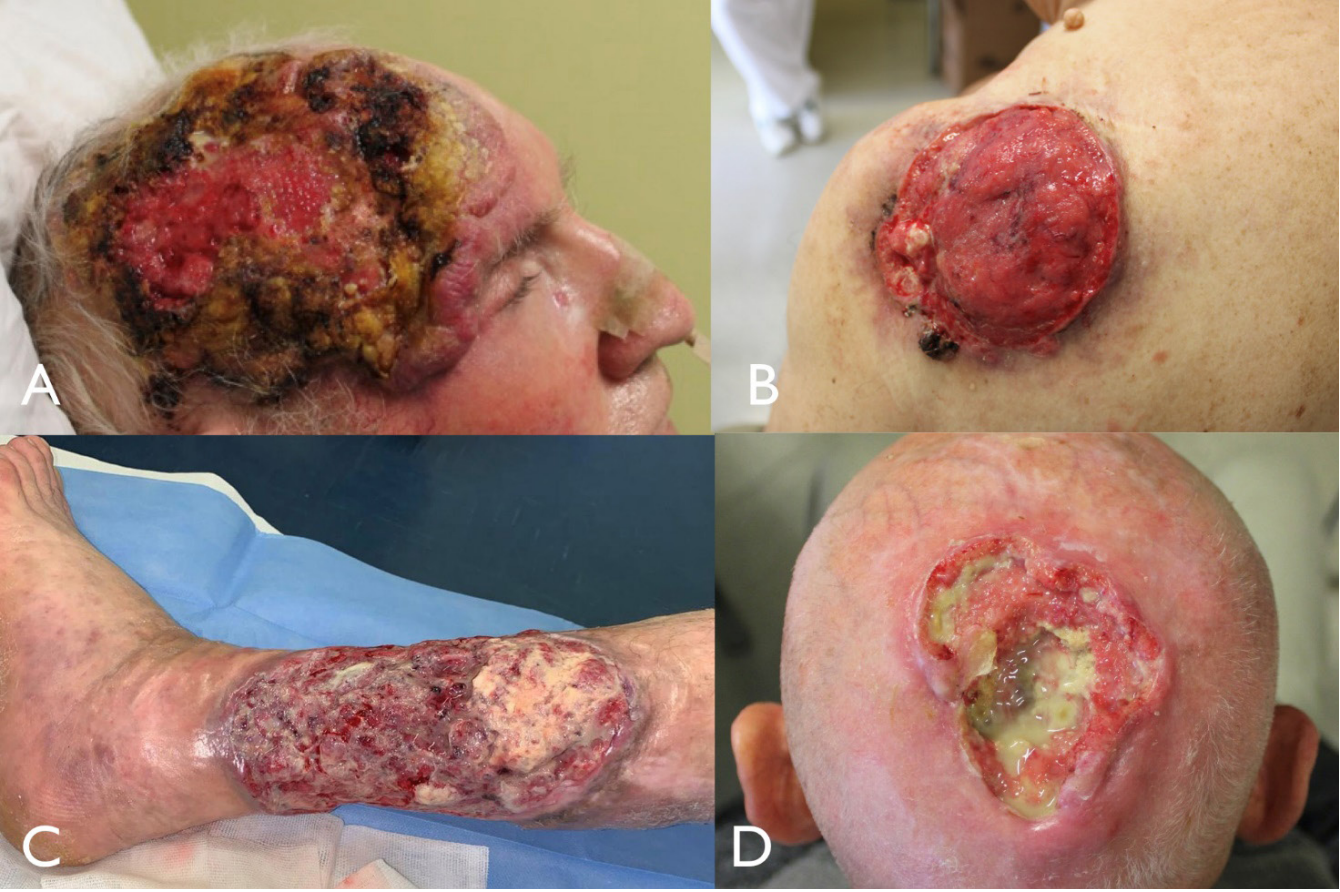
Locally advanced cutaneous squamous cell carcinoma (cSCC). (A) cSCC in a 83-year-old male patient, located on the right parietal region, no previous treatment, late diagnosis. (B) cSCC in a 77-year-old male patient on the left shoulder, recurrent to surgery. (C) cSCC on the leg of a 61-year-old immunosuppressed male, no previous treatment. (D) cSCC on the scalp of a 89-year-old male patient, recurrent after radiotherapy.

Locally advanced cSCC has been defined as non-metastatic cSCC that is unlikely to be cured with surgery, radiotherapy, or combination treatment (surgery and radiotherapy) (Figure 2). However, locally advanced disease lacks consensus definition and diagnosis is made after multidisciplinary board consultation. Thus, the diagnosis of locally advanced cSCC is influenced by the expertise of each center. The need for standardized definition criteria of locally advanced cSCC is high in the clinical trial setting.

In phase 2 trial of cemiplimab for advanced cSCC, locally advanced cSCC patients were included if they experienced recurrence after 2 or more surgical procedures with subsequent unlikely curative resection, if the tumor already reached substantial local invasion precluding complete resection, or if surgical treatment would lead to substantial complications or deformity. Acceptable reasons for RT to be considered inappropriate were: previous RT with further RT exceeding the threshold of an acceptable cumulative dose, judgment of the radiation oncologist that the tumor was unlikely to respond to RT, or a risk–benefit assessment that RT was contraindicated for the patient [30].

Clinical progression of cSCC into the advanced form seems to be associated not only to the intrinsic aggressiveness of cSCC, but also to patient characteristics that may impact on clinician decision and incomplete tumor initial management.

Regarding real-life profile of advanced cSCC patients, a retrospective study by Hillen et al [3], analyzed 190 patients with advanced cSCC. Patients presented a median age of 78 years, an ECOG status 0–1, and location of the primary tumor most frequently on the head and neck, including high-risk locations such as ears or lips. Despite nonmalignant comorbidities influenced the decision for cSCC-specific therapy in only 21% of patients, the authors highlighted the fact that many clinicians might be unaware that locally advanced cSCC can lead to death. Eigentler et al [31], showed that in a cohort of 1,434 patients who excised a cSCC between 2005 and 2015 and were followed-up for a median period of 36.5 months, a higher number of patients died due to local infiltration in the head region or regional infiltration into neck lymph nodes, compared to death due to visceral metastases.

Concerning pitfalls of initial management of cSCC, a retrospective study by Deilhes et al [32], in a cohort of 109 patients with advanced cSCC, showed that 63% of patients had a delay of more than 3 months between the lesion’s first observation and biopsy, 62% of patients had incomplete histological examinations, and only 35% of patients completed all the procedures required for optimal management of the disease. Moreover, the authors highlighted that 75% of their patients’ cohort were living in rural areas and the decreased availability of dermatologists might have impacted mismanagement of the disease.

## Conclusion

The clinician’s goal should be the recognition and appropriate treatment of cSCC at higher risk for recurrence and/or for progressing into advanced disease. However, it should be acknowledged that diagnosis, management, and follow-up of high-risk cSCC are still not straightforward. More studies are needed to standardize the relevance of each risk/prognostic factor, to explore the risk estimation of outcomes, and to prove the utility of disease-staging modalities. The adjuvant setting should be further explored to prevent progression of the disease. Currently, diagnosis and management of high-risk and advanced cSCC rely on a multidisciplinary approach that favors the most suitable therapeutic option based on the characteristics of the patient and of the disease.

## Figures and Tables

**Table 1:** TNM Clinical Classification for Skin Carcinoma (excluding eyelid, head and neck, perineal, vulva and penis) According to UICC 8th Edition

**T – Primary Tumor**	
Tx	Primary tumor cannot be identified
T0	No evidence of primary tumor
Tis	Carcinoma in situ
T1	Tumor 2 cm or less in greatest dimension
T2	Tumor > 2 cm and ≤4 cm in greatest dimension
T3	Tumor > 4 cm in greatest dimension *or* minor bone erosion *or* perineural invasion (clinical or radiographic involvement of named nerves without foramen or skull base invasion or transgression*) *or* deep invasion (invasion beyond the subcutaneous fat or > 6 mm measured from the granular layer of adjacent normal epidermis to the base of the tumor)
T4a	Tumor with gross cortical bone/marrow invasion
T4b	Tumor with axial skeleton invasion including foraminal involvement and/or vertebral foramen involvement to the epidural space
Nb. In the case of multiple simultaneous tumors, the tumor with the highest T category is classified and the number of separate tumors is indicated in parentheses, eg T2(5). *In AJCC staging, perineural invasion is defined, above this definition, also as “tumor cells within the nerve sheath of a nerve lying deeper than the dermis or measuring 0.1 mm or larger in caliber”	
**N – Regional Lymph Nodes**	
Nx	Regional lymph nodes cannot be assessed
N0	No regional lymph node metastasis
N1	Metastasis in a single lymph node 3 cm or less in greatest dimension
N2	Metastasis in a single ipsilateral lymph node, more than 3 cm but no more than 6 cm in greatest dimension or in multiple ipsilateral lymph nodes none more than 6 cm in greatest dimension
N3	Metastasis in a lymph node more than 6 cm in greatest dimension
**M – Distant Metastasis**	
M0	No distant metastasis
M1	Distant metastasis (comprising contralateral nodes)

**Table 2:** pTNM Pathological Classification for Skin Carcinoma (excluding eyelid, head and neck, perineal, vulva and penis) According to UICC 8^th^ Edition

The pT and pN categories correspond to the clinical T and N categories	
**pN0**	Histological examination of a regional lymphadenectomy specimen will ordinarily include 6 or more lymph nodes. If the lymph nodes are negative, but the number of ordinarily is not met, classify as pN0.
**pM1**	Distant metastasis microscopically confirmed.

**Table 3:** TNM Clinical Classification for Skin Carcinoma of the Head and Neck According to AJCC/UICC 8^th^ Edition

**T – Primary Tumor**	
Tx	Primary tumor cannot be identified
T0	No evidence of primary tumor
Tis	Carcinoma in situ
T1	Tumor 2 cm or less in greatest dimension
T2	Tumor > 2 cm and ≤4 cm in greatest dimension
T3	Tumor > 4 cm in greatest dimension *or* minor bone erosion *or* perineural invasion (clinical or radiographic involvement of named nerves without foramen or skull base invasion or transgression*) *or* deep invasion (invasion beyond the subcutaneous fat or > 6 mm measured from the granular layer of adjacent normal epidermis to the base of the tumor)
T4a	Tumor with gross cortical bone/marrow invasion
T4b	Tumor with skull base or axial skeleton invasion including foraminal involvement and/or vertebral foramen involvement to the epidural space
Nb. In the case of multiple simultaneous tumors, the tumor with the highest T category is classified and the number of separate tumors is indicated in parentheses, eg T2(5). * In AJCC staging, perineural invasion is defined, above this definition, also as “tumor cells within the nerve sheath of a nerve lying deeper than the dermis or measuring 0.1 mm or larger in caliber”	
**N – Regional Lymph Nodes**	
N0	No regional lymph node metastasis
N1	Metastasis in a single ipsilateral lymph node, 3 cm or less in greatest dimension without extranodal extension
N2a	Metastasis in a single ipsilateral lymph node, more than 3 cm but no more than 6 cm in greatest dimension without extranodal extension
N2b	Metastasis in multiple ipsilateral lymph nodes, none more than 6 cm in greatest dimension without extranodal extension
N2c	Metastasis in bilateral or controlateral lymph nodes, none more than 6 cm in greatest dimension, without extranodal extension
N3a	Metastasis in a lymph node more than 6 cm in greatest dimension without extranodal extension
N3b	Metastasis in a single or multiple lymph nodes with clinical extranodal extension (defined as the presence of skin involvement or soft tissue invasion with deep fixation/tethering to underlying muscle or adjacent structures or clinical signs of nerve involvement)
**M – Distant Metastasis**	
M0	No distant metastasis
M1	Distant metastasis (comprising contralateral nodes)

**Table 4:** pTNM Pathological Classification for Skin Carcinoma of the Head and Neck according to UICC 8^th^ Edition

The pT categories correspond to the clinical T categories	
**pN – Regional Lymph Nodes**	
Histological examination of a selective neck dissection specimen will ordinarily include 10 or more lymph nodes. Histological examination of a radical or modified radical dissection specimen will ordinarily include 15 or more lymph nodes.	
pNx	Regional lymph nodes cannot be assessed
pN0	No regional lymph node metastasis
pN1	Metastasis in a single ipsilateral lymph node, 3 cm or less in greatest dimension without extranodal extension
pN2a	Metastasis in a single ipsilateral lymph node, less than 3 cm in greatest dimension with extranodal extension, or more than 3 cm but not more than 6 cm in greatest dimension without extranodal extension
pN2b	Metastasis in multiple ipsilateral lymph nodes, none more than 6 cm in greatest dimension, without extranodal extension
pN2c	Metastasis in bilateral or controlateral lymph nodes, none more than 6 cm in greatest dimension, without extranodal extension
pN3a	Metastasis in a lymph node more than 6 cm in greatest dimension without extranodal extension
pN3b	Metastasis in a lymph node more than 3 cm in greatest dimension with extranodal extension or multiple ipsilateral, or any contralateral or bilateral node(s) with extranodal extension
**pM – Distant Metastasis**	
pM1	Distant metastasis microscopically confirmed

**Table 5:** Staging System for Both Skin Carcinoma (excluding eyelid, head and neck, perineal, vulva and penis) and Skin Carcinoma of the Head and Neck According to AJCC/UICC 8^th^ Edition

Stage 0	Tis	N0	M0
Stage I	T1	N0	M0
Stage II	T2	N0	M0
Stage III	T3	N0	M0
	T1, T2, T3	N1	M0
Stage IVA	T1, T2, T3	N2, N3	M0
	T4	Any N	M0
Stage IVB	Any T	Any N	M1

**Table 6:** Prognostic Factors in cSCC According to the EADO and NCCN Guidelines. Definition of High-Risk Patients (and very high-risk patients in the NCCN classification)

	EADO	NCCN
**Intrinsic factors**		
**Size**	> 2 cm	High-risk: > 2 and < 4 cm Very high-risk: > 4 cm
**Location**	Temple, ear, lip	Head, neck, hands, feet, pretibial, anogenital area
**Depth of invasion**	> 6 mm or beyond fat tissue	> 6 mm or beyond fat tissue
**Perineural invasion**	Microscopic, symptomatic or radiological	High-risk: + Very high-risk: Tumor cell within the nerve sheath of a nerve lying deeper than the dermis or measuring ≥ 0.1 mm
**Degree of differentiation**	Poor differentiation	Poor differentiation
**Desmoplasia**	+	Very high-risk: + Other subtypes for high-risk: Acantholytic, adenosquamous, metaplastic
**Growth rate**	-	+ Rapidly growing tumor
**Bone erosion**	+	-
**Borders**	-	+ poorly defined
**Lymphatic or vascular involvement**	-	High-risk: - Very high-risk: +
**Extrinsic factors**		
**Primary vs recurrent**	-	Recurrent
**Prior radiotherapy**	-	+
**Immunosuppression**	+	+
